# Total Phenolic and Flavonoid Content and Biological Activities of Extracts and Isolated Compounds of *Cytisus villosus* Pourr.

**DOI:** 10.3390/biom9110732

**Published:** 2019-11-13

**Authors:** Farida Larit, Francisco León, Samira Benyahia, Stephen J. Cutler

**Affiliations:** 1 Department of BioMolecular Sciences, School of Pharmacy, University of Mississippi, University, MS 38677-1848, USA; JLeonOyola@cop.ufl.edu (F.L.); SJCUTLER@cop.sc.edu (S.J.C.); 2 Département de Chimie, Faculté des Sciences Exactes, Université des Frères Mentouri Constantine 1, Constantine, Route d’Aine El Bey, 25000 Constantine, Algeria; 3 Department of Medicinal Chemistry, College of Pharmacy, University of Florida, Gainesville, FL 32610, USA; 4 Laboratoire de Synthèse Organique, Modélisation et Optimisation des Procèdes (LOMOP), Université Badji Mokhtar, Faculté des Sciences, Département de Chimie, 23000 Annaba, Algeria; samira.benyahia13@gmail.com; 5 College of Pharmacy, University of South Carolina, Columbia, SC 29208, USA

**Keywords:** *Cytisus villosus*, antioxidant, anti-inflammatory, antibacterial, antifungal, antimalarial, antileishmanial, antitrypanosomal, cytotoxicity, cannabinoid receptor, opioid receptor

## Abstract

The aim of this study was to evaluate the total phenolic and flavonoid content, and the in vitro antioxidant, anti-inflammatory, antibacterial, antifungal, antimalarial, cytotoxicity, and antiprotozoal activities of the Algerian plant *Cytisus villosus* Pourr. (Syn. *Cytisus triflorus* L’Hérit.). Additionally, the radioligand displacement affinity on opioid and cannabinoid receptors was assessed for the extracts and isolated pure compounds. The hydro alcoholic extract of the aerial part of *C. villosus* was partitioned with chloroform (CHCl_3_), ethyl acetate (EtOAc), and *n*-butanol (*n*-BuOH). The phenolic content of the *C. villosus* extracts was evaluated using a modified Folin–Ciocalteau method. The total flavonoid content was measured spectrometrically using the aluminum chloride colorimetric assay. The known flavonoids genistein (**1**), chrysin (**2**), chrysin-7-*O*-β-d-glucopyranoside (**3**), and 2″-*O*-α-l-rhamnosylorientin (**4**) were isolated. The antioxidant activities of the extracts and isolated compounds were evaluated using 2,2-diphenyl-1-picrylhydrazyl (DDPH) and cellular antioxidant activity (CAA) assays. The plant extracts showed moderate antioxidant activity. EtOAc and *n*-BuOH extracts showed moderate anti-inflammatory activity through the inhibition of induced nitric oxide synthase (iNOS) with IC_50_ values of 48 and 90 µg/mL, respectively. The isolated pure compounds 1 and 3 showed good inhibition of Inducible nitric oxide synthase (iNOS) with IC_50_ values of 9 and 20 µg/mL, respectively. Compounds **1** and **2** exhibited lower inhibition of Nuclear Factor kappa-light-chain-enhancer of activated B cells (NF-κB) with IC_50_ values of 28 and 38 µg/mL, respectively. Furthermore, the extracts and isolated pure compounds have been shown to exhibit low affinity for cannabinoid and opioid receptors. Finally, *n*-BuOH extract was a potent inhibitor of *Trypanosoma brucei* with IC_50_ value of 7.99 µg/mL and IC_90_ value of 12.61 µg/mL. The extracts and isolated compounds showed no antimicrobial, antimalarial nor antileishmanial activities. No cytotoxic effect was observed on cancer cell lines. The results highlight this species as a promising source of anti-inflammatory and antitrypanosomal agents.

## 1. Introduction

Natural compounds derived from plants have played an important role from ancient to recent times in the management and treatment of many maladies with wide effects, such as antioxidants associated with reduced risks of cancer, cardiovascular disease, diabetes, infectious diseases, and other disorders associated with age [1]. The advantage of many natural products, which have been components of the human diet for several thousand years, is that the human organism has become adapted to them, which may decrease the risk of harmful side effects.

Polyphenols are a major class of natural compounds of medicinal importance, exhibiting a wide range of biological and pharmacological activities, such as antioxidant, anti-inflammatory, immunostimulant, anti-aging, antitumor, antidepressant, and antiparasitic [2,3]. The high antioxidant activity of polyphenols is mainly due to their redox properties, which allow them to act as reducing agents, hydrogen donors, and singlet oxygen quenchers. In this context, oxidative stress plays an important role in the progression of neurodegenerative conditions, including rheumatic and cardiovascular disorders, metabolic syndrome, and other diseases [4]. Inflammation is considered to be a risk factor for hypertension, diabetes, and several types of cancer, and can be involved in Alzheimer’s disease pathogenesis.

Nuclear factor-kappa B (NF-κB), inducible nitric oxide synthase (iNOS), and reactive oxygen species (ROS) have long been considered as important targets for new anti-inflammatory drugs. NF-κB plays a central role in inflammation through its ability to induce transcription of proinflammatory genes, hence, NF-κB has been implicated in the pathogenesis of many inflammatory and age-associated diseases [5]. NF-κB complex proteins are widely expressed in the developing and mature nervous system. The effects of NF-κB on neurons have been widely investigated and, recently, it has been reported that the NF-κB family of transcription factors has a major role in regulating the growth and elaboration of neural processes [6]. Furthermore, NF-κB has been found to play a role in enhancing neuronal apoptosis associated with ischemic brain injury, neurodegenerative diseases, and inflammatory conditions [7,8]. In fact, Bonini et al. [9] demonstrated that there are potential links between the altered function of the NF-κB pathway and pathogenesis of neurodevelopmental disorders [9]. Excessive generation of nitric oxide (NO) and ROS contribute significantly to the progress of inflammation [10]. Inhibition of iNOS can reduce the intracellular NO production [11].

Infections caused by several protozoa microorganism, including *Trypanosoma*, *Plasmodium,* and *Leishmania*, are a major worldwide health problem causing significant morbidity and mortality in Africa, Asia, and South America. According to the World Health Organization (WHO) statistics, there are 12 million people currently affected by leishmaniasis in 88 countries, including Algeria with 350 million people at risk [12]. Current available drugs for the treatment of these infections suffer from high toxicities, which may cause serious side effects. Thus, there is an urgent need to develop safer and more efficient compounds for the treatment of these diseases. Polyphenols, specifically flavonoids, have been known to be important resources to find new antiprotozoal, non-toxic drug candidates [13,14].

Several herbs used in folk medicine have been suggested as important sources for treatment of depression, Alzheimer’s and Parkinson’s diseases, and other neuropsychiatric as well as neurological disorders [15,16,17]. The interaction of medicinal plants with central nervous system (CNS) receptors is well reported [18,19,20]. Specifically, the opioid system has been described to play different roles in inflammation, the cancer process, and to be a potential target for therapy of various neurological disorders [21,22,23]. The case of cannabinoid receptors (CB1 and CB2) has gained much attention as potential pharmacotherapeutic targets to control some CNS disorders, in particular those related to neuroinflammatory and neurodegenerative events, such as Alzheimer’s disease (AD) [24,25]. Within the last decade, medicine based on opioids and cannabinoids has found many applications, including as anti-inflammatory agents and analgesics [26,27].

*Cytisus* (Fabaceae) is a large and diversified genus, including approximately 60 species, which are particularly abundant around the Mediterranean Sea [28]. Plants of this genus have been used in folk medicine as a diuretic and in the treatment of mild hypertension, heart failure, cardiac edema, and wounds. *Cytisus* species have been found to exhibit bioactive properties, including antioxidant, anti-inflammatory, anxiolytic, antiparasitic, and antidiabetic activities [29,30,31]. The therapeutic properties of *Cytisus* are related to their high concentration of phenolic compounds, including flavonoids and caffeic acids [32]. In continuation of previous works on Algerian plants [33,34], herein, we extended our study to evaluate the antioxidant, anti-inflammatory, antiprotozoal, antimalarial, antimicrobial, cytotoxicity, and radioligand displacement affinity on opioid and cannabinoid receptors activities of extracts and isolated pure compounds of *Cytisus villosus* Pourr. (Syn. *Cytisus triflorus* L’Hérit.).

## 2. Materials and Methods

### 2.1. General Experimental Procedures

UV spectra were obtained using a Perkin-Elmer Lambda 3B UV/vis-spectrophotometer (Perkin Elmer Inc, Waltham MA, USA). ^1^H and ^13^C NMR spectra were obtained using Bruker model AMX 500 and 400 NMR spectrometers with standard pulse sequences, operating at 500 and 400 MHz in ^1^H and 125 and 100 MHz in ^13^C, respectively. Coupling constants were recorded in Hertz (Hz). Standard pulse sequences were used for Heteronuclear and homonuclear 2D NMR experiments. All spectra were run at 25 °C. High-resolution mass spectra (HRMS) (Bruker Corporation, Billerica MA, USA) were measured on a Micromass Q-Tof Micro mass spectrometer with a lock spray source (Waters Corporation, Milford MA, USA). Column chromatography was carried out on silica gel (70–230 mesh, Merck, Darmstadt, Germany), C18 Solid Phase extraction (SPE) (500 mg Bed, Thermo scientific INC, Waltham MA, USA), Diaion HP-20 (Sorbetch technologies, Norcross GA, USA), and sephadex LH-20 (Sorbetch technologies Norcross GA, USA USA). Thin Layer Chromatography (TLC) (silica gel 60 F254, Merck, Darmstadt, Germany) was used to monitor fractions from column chromatography. Preparative TLC was carried out on silica gel 60 PF254+366 plates (20 × 20 cm, 1 mm thick). Visualization of the TLC plates was achieved with a UV lamp (λ = 254 and 365 nm) and anisaldehyde/acid spray reagent (MeOH-acetic acid-anisaldehyde-sulfuric acid, 85:9:1:5).

### 2.2. Plant Material

The aerial parts of *Cytisus villosus* Pourr. were collected from the Collo region, in Northeastern Algeria during its flowering stage in April 2010. A voucher specimen (UM-10232015) has been deposited in the culture collection of the Department of BioMolecular Sciences, University of Mississippi.

### 2.3. Extraction and Isolation

Dried powdered aerial parts of *C. villosus* (1 kg) were macerated at room temperature with EtOH–H_2_O (80:20, *v/v*) for 24 h, three times. The filtered crude extracts were combined and concentrated under reduced pressure to afford 25 g of extract, which was suspended in distilled water (800 mL) and successively partitioned with chloroform (CHCl_3_), ethyl acetate (EtOAc), and n-butanol (*n*-but), yielding 500 mg (CHCl_3_), 5 g (EtOAc), and 10 g (*n*-butanol) fractions, respectively. The ethyl acetate fraction (5 g) was subjected to silica gel column, eluted initially with CH_2_Cl_2_: MeOH (95:5) and then gradient eluted with CH_2_Cl_2_: MeOH at ratios 90:10, 85:15, 80:20, 50:50, 20:80, and finally with 100% MeOH. Each subfraction was monitored by TLC on silica gel using CHCl_3_:EtOAc:HCOOH (5:4:1) and CH_2_Cl_2_:MeOH (1:1) systems. Similar subfractions were combined together and concentrated under reduced pressure to yield seven main subfractions (I to VII). Subfraction II (170 mg) was subjected to Sephadex LH-20 column using MeOH as the solvent to afford compound 1 (5 mg, genistein) as light-yellow needles. Subfraction III (161 mg) was subjected to Sephadex LH-20 using MeOH as an eluent to yield compound 2 (4 mg) as a yellow amorphous powder. Subfraction V (250 mg) was subjected to Sephadex LH-20 using MeOH:CH_2_Cl_2_ (1:1) as an eluent to give compound 3 (3 mg) as a yellowish amorphous powder. The *n*-BuOH fraction (10 g) was subjected to Diaion HP-20 column chromatography and eluted with distilled H_2_O then MeOH to give two main subfractions, the aqueous subfraction A (6 g) and the methanolic subfraction M (4 g). The methanolic subfraction M (4 g) was subjected to MN-polyamide-SC-6 (150 g) column chromatography which was eluted with water and then with water-methanol systems gradient decreased polarities to afford eight subfractions (M-1 to M-8). Subfraction M-3 (250 mg) was rechromatographed on Sephadex LH-20 column eluted with MeOH:CH_2_Cl_2_ (1:1) to yield compound 4 (6 mg) as yellow crystals.

### 2.4. Determination of Total Phenolic and Total Flavonoid Content 

Folin–Ciocalteu reagent, gallic acid, and quercetin standards were obtained from Sigma-Aldrich (Poznan, Poland). Aluminum chloride hexahydrate, methanol, and sodium carbonate were obtained from Sigma-Aldrich (Poznan, Poland). The total phenolic was measured using spectrophotometry with a modified Folin–Ciocalteu method [35]. Total phenol content, expressed as milligrams of gallic acid equivalent (GAE) per gram of extract (GAE mg/g), was calculated on the basis of a standard calibration curve of gallic acid (Y = 0.1157*x* + 0.087, *R*^2^ = 0.9749). Total flavonoid content of the plants fractions crud extracts was determined by colorimetric method [36,37]. The concentration of total flavonoid content in the test samples was calculated from the calibration plot (Y = 1.2308*x* + 0.0151, *R*^2^ = 0.9775) and expressed as mg quercetin equivalent (QE)/g of dried extract. The extracts were dissolved in dimethyl sulfoxide (DMSO) to make a stock solution of 20 mg/mL.

### 2.5. Antioxidant Activity 

#### 2.5.1. Diphenyl-1-picrylhydrazyl (DPPH) Assay

The antioxidant activity of the extracts and pure isolated compounds was determined by applying the 2,2-diphenyl-1-picrylhydrazyl (DPPH) radical scavenging method [38].

#### 2.5.2. Cellular Antioxidant Activity (CAA) Assay 

The cellular antioxidant activity was measured in HepG2 cells as described by Wolfe and Rui [39,40]. The antioxidant activity was expressed in terms of CAA units. The area under the curve (AUC) of fluorescence versus time plot was used to calculate CAA units as described by Wolfe and Rui [39,40].

### 2.6. Anti-Inflammatory Activity

#### 2.6.1. Anti-Inflammatory Activity Assay for the Inhibition of iNOS 

The extracts and isolated compounds of *C. villosus* were evaluated in terms of their interaction with cellular targets related to inflammation and metabolic disorders, such as iNOS and NF-κB. The inhibition of intracellular NO production as a result of iNOS activity was assayed in mouse macrophages (RAW 264.7cells) [41]. Cytotoxicity of test samples to macrophages was also determined in parallel to check if the inhibition of iNOS was due to cytotoxic effects.

#### 2.6.2. Reporter Gene Assay for the Inhibition of NF-κB 

Reporter gene assay for the inhibition of NF-κB Activity was performed as described earlier [42]. In brief, cells transfected with NF-κB luciferase plasmid construct were plated in 96-well plates at a density of 1.25 × 105 cells/well. After 24 h, cells were treated with the test compounds and, after incubating for 30 min, phorbol 12-myristate 13-acetate (PMA) (Sigma-Aldrich, Burlington MA, USA) (70 ng/mL) was added and further incubated for 6−8 h. Luciferase activity was measured as described above. Percent decrease in luciferase activity was calculated relative to the vehicle control. Parthenolide (Sigma-Aldrich, Burlington MA, USA) was used as a positive control.

### 2.7. Antiprotozoal Assay

The in vitro antileishmanial and antitrypanosomal assays were done on cell cultures of *L. donovani* promastigotes, axenic amastigotes, THP1-amastigotes, and *Trypanosoma brucei* trypomastigotes by Alamar Blue assays [43]. The conditions for seeding the THP1 cells, exposure to the test samples, and evaluation of cytotoxicity were the same as described in parasite-rescue and transformation assay [44]. IC_50_ and IC_90_ values were computed from the dose response curves using XLfit software (XLfit 5.3.1, IDBS analytical, Boston MA, USA). DFMO (difluoromethylornithine) was used as the positive control. The antiprotozoal activity of *C. villosus* extracts and isolated compounds were evaluated in vitro against *L. donovani* promastigotes, axenic amastigotes, and intracellular amastigotes in THP1 cells. The extracts and some isolated compounds were also evaluated against *T. brucei* trypomastigote forms. All the extracts and compounds were simultaneously tested against THP1 cell for determination of general cytotoxicity. The extracts and isolated compounds were also evaluated for their antimalarial activity against chloroquine-sensitive (D6, Sierra Leone) and chloroquine-resistant strains (W2, Indochina) strains of *Plasmodium falciparum* [45]. Furthermore, they were tested for cytotoxicity against the Vero cell line.

### 2.8. Antimicrobial Assay

Extracts and pure compounds were tested for their antimicrobial activity against *Staphylococcus aureus*, methicillin-resistant *S. aureus* (MRSA), *Escherichia coli*, *Pseudomonas aeruginosa*, and *Mycobacterium intracellulare*. The antifungal activities were evaluated against a panel of pathogenic fungi, including *Candida albicans*, *C. glabrata*, *C. krusei*, *Aspergillus fumigatus*, and *Cryptococcus neoformans*, associated with opportunistic infections. Ciprofloxacin (MP Biomedicals Inc, Aurora OH, USA) for antibacterial bioassays and Amphotericin B (MP Biomedicals Inc, Aurora OH, USA) for fungal bioassays were used as positive controls, respectively [45].

### 2.9. Cytotoxicity Assays 

Each assay was performed in 96-well tissue culture-treated microplates. Cytotoxic activity was determined against four human cancer cell lines (SK-MEL, KB, BT-549, andSKOV-3,) and two noncancerous kidney cell lines (LLC-PK1 and Vero). All cell lines were obtained from the American Type Culture Collection (ATCC, Rockville, MD, USA). Each assay was performed in 96-well tissue culture-treated microplates [46]. Cells were seeded at a density of 25,000 cells/well and incubated for 24 h. Samples at different concentrations were added and cells were again incubated for 48 h. At the end of incubation, the cell viability was determined using neutral red dye according to a modification of the procedure of Borenfreund et al. [46,47]. IC_50_ values were determined from dose−response curves of percent growth inhibition against test concentrations. Doxorubicin was used as a positive control, while DMSO was used as the negative (vehicle) control.

### 2.10. Radioligand Displacement for Cannabinoid and Opioid Receptor Subtypes

The evaluated extracts and isolated compounds of *C. villosus* were run in competition binding with cannabinoid receptor subtypes, cannabinoid receptor 1 (CB_1_) and cannabinoid receptor 2 (CB_2_), and were tested against the opioid receptor subtypes (μ, κ, and δ) as previously described [48].

### 2.11. Statistical Analysis 

All the experiments for determination of total phenolics, total flavonoids, and antioxidant properties using DPPH and cellular antioxidant assay (CAA) were conducted in triplicates. The values are expressed as the mean ± standard deviation (SD). Analysis of variance and significance of difference among means were tested by one-way ANOVA and least significant difference (LSD) on mean values. Correlation coefficients (*R*) and coefficients of determination (*R*^2^) were calculated using Microsoft Excel 2007.

## 3. Results

### 3.1. Chemistry

Phytochemical study of the hydro ethanolic extract of the aerial part of *C. villosus* led to the isolation of four known flavonoids (**1**) genistein, (**2**) chrysin, (**3**) chrysin-7-*O*-β-d-glucopyranoside, and (**4**) 2″-*O*-α-l-rhamnosylorientin (Figure 1). The structures of the known compounds were identified by comparison of their spectroscopic data with those reported in the literature [33]. The spectroscopic data for the isolated compounds can be seen the Supplementary Materials Figures S1–S12.

### 3.2. Determination of Total Phenolic and Total Flavonoid Contents

Table 1 shows the total phenolic content in the extracts of the *C. villosus* aerial parts. Total phenolic content was measured for the CHCl_3_, EtOAc, and *n*-BuOH extracts. Among the extracts of *C. villosus*, the highest phenolic content was found in the *n*-BuOH extract (363.00mg GAE/g dried extract) followed by EtOAc (208.00 mg GAE/g dried extract) and CHCl_3_ extract (56.00 mg GAE/g dried extract). The total flavonoids content in the *C. villosus* extracts are shown in Table 1. Similarly, the highest amount of flavonoid content was found in the *n*-BuOH extract (21.16 mg QE/g dried extract).

### 3.3. Determination of Antioxidant Activity

#### 3.3.1. 2,2-Diphenyl-1-picrylhydrazyl (DPPH) Assay

**Figure 2:**
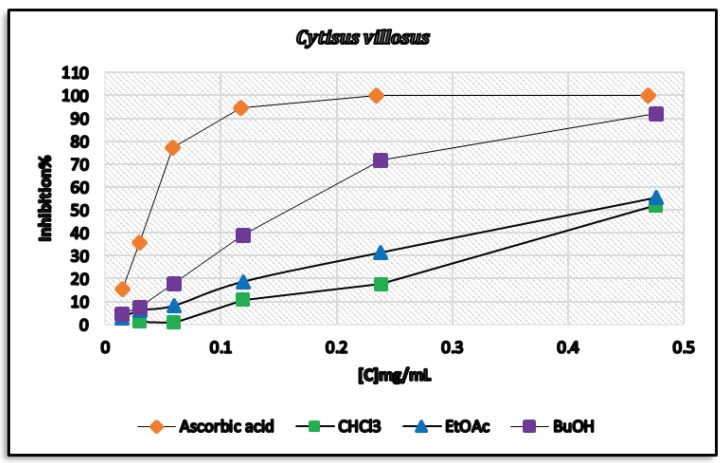
Radical scavenging effect of *C. villosus* extracts on 2,2-Diphenyl-1-picrylhydrazyl (DPPH) radical. Each value is represented as mean ±SD.

The antioxidant activity of extracts and isolated compounds of *C. villosus* was evaluated in terms of their free radical scavenging capacity (DPPH) assay (Figure 2). The CHCl_3_ and EtOAc extracts of *C. villosus* showed moderate antioxidant activity with IC_50_ values of 0.459 and 0.425 mg/mL, respectively. The *n*-BuOH extract showed highly antioxidant activity against DDPH compared to EtOAc and CHCl_3_ extracts with an IC_50_ value of 0.164 mg/mL (Table 1).

#### 3.3.2. Cellular Antioxidant Activity (CAA) assay

The antioxidant activity of *C. villosus* extracts and isolated pure compounds was also evaluated using the cellular antioxidant assay (CAA). The results are shown in Table 2. The extracts of *C. villosus* showed weak inhibition of intracellular oxidative stress (29% to 36% inhibition of ROS generation at 250 μg/mL). Similarly, the tested isolated compounds were not effective except for compound 4 from *n*-BuOH extract (Figure 2). Compound 4 showed weak inhibition of intracellular oxidative stress (28% at 250 µg/mL) (Table 2).

### 3.4. Determination of Anti-Inflammatory Activity 

The EtOAc and *n*-BuOH extracts of *C. villosus* showed weak inhibition of iNOS with IC_50_ values of 48 and 90 µg/mL, respectively. Compounds 1 and 3 isolated from the EtOAc extract of *C. villosus* showed good inhibition of iNOS with IC_50_ values of 9 and 20 µg/mL, respectively (Table 3). The increase in transcriptional activity of NF-κB in PMA-treated cells was also not suppressed by the plant’s extracts and isolated compounds with the exception of compounds 1 and 2, which showed moderate inhibition of NF-κB activity with IC_50_ values of 28 and 38 µg/mL, respectively (Table 3).

### 3.5. Antiprotozoal Activity 

The results for this assay are presented in Table 4. The EtOAc extract showed weak antitrypanosomal activity against *T. brucei* with IC_50_ values of 19.48 µg/mL, while the *n*-BuOH extract was found to exhibit high antitrypanosomal activity against *T. brucei* with IC_50_ values of 7.99 µg/mL and IC_90_ values of 12.61 µg/mL. No significant activity was observed in vitro against *Leishmania donovani* (promastigotes, axenic amastigotes, and intracellular amastigotes in THP1 cells).

### 3.6. Antimicrobial Activity

The plant’s extracts and isolated compounds showed no antimicrobial activity against all tested microorganisms. The results of antimicrobial assay are given in Table 5.

### 3.7. Anti-Malarial Activity

The results of the antimalarial activity assay are presented in Table 6 and Table 7. No antimalarial activity was observed against chloroquine-sensitive and chloroquine-resistant strains of *Plasmodium*.

### 3.8. Cytotoxicity

The results of Cytotoxicity assays are shown in Table 8. The tested extracts and isolated compounds of *C. villosus* were not active against any cell lines used in this study.

### 3.9. Radioligand Displacement for Cannabinoid and Opioid Receptor Subtypes

The affinity of the total extracts and isolated compounds towards cannabinoid and opioid receptors was tested. The results are shown in Table 9. Low affinity for cannabinoids was found in both extracts evaluated and no affinity for the compound 2. For opioids, both fractions tested revealed a preference toward δ-opioids with low displacement values.

## 4. Discussion

The antioxidant capacity of medicinal plants extracts and pure natural compounds can be tested using various methods. In the present study, the antioxidant activity of the studied species extracts and its isolated phenolics were evaluated in terms of their free radical scavenging capacity by DPPH assay. Their activity against intracellular oxidative stress was determined by CAA assay. Our results showed that the radical scavenging activity of the *n*-BuOH extract of *C. villosus* aerial parts was high compared to the EtOAc and CHCl_3_ extracts. The *n*-BuOH extract was found to have the highest inhibition of intracellular oxidative stress with 36% inhibition at 250 μg/mL.

**Figure 3:**
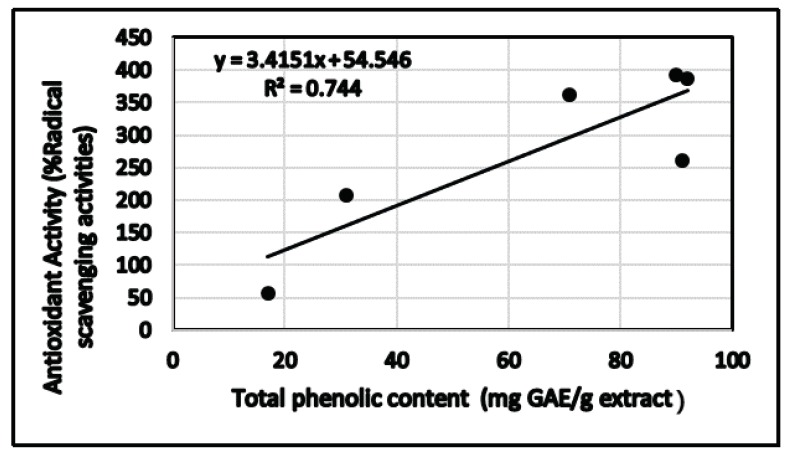
Correlation graphs for DPPH (% radical scavenging activity) and total phenolic content in the *C. villosus* extracts.

**Figure 4:**
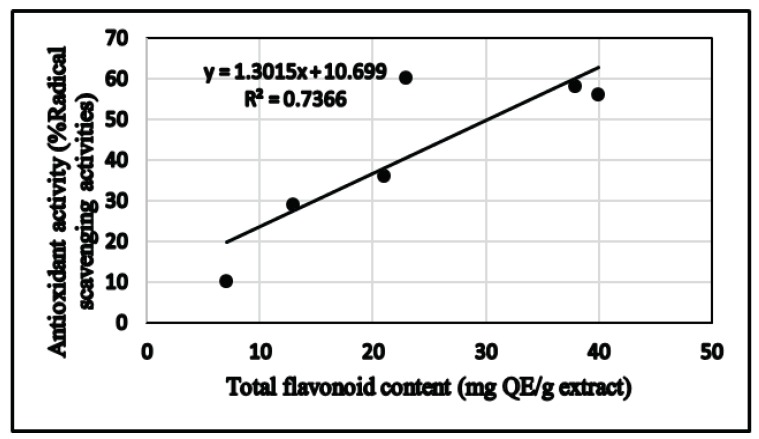
Correlation graphs for DPPH (% radical scavenging activity) and total flavonoid content in the *C. villosus* extracts.

**Figure 5:**
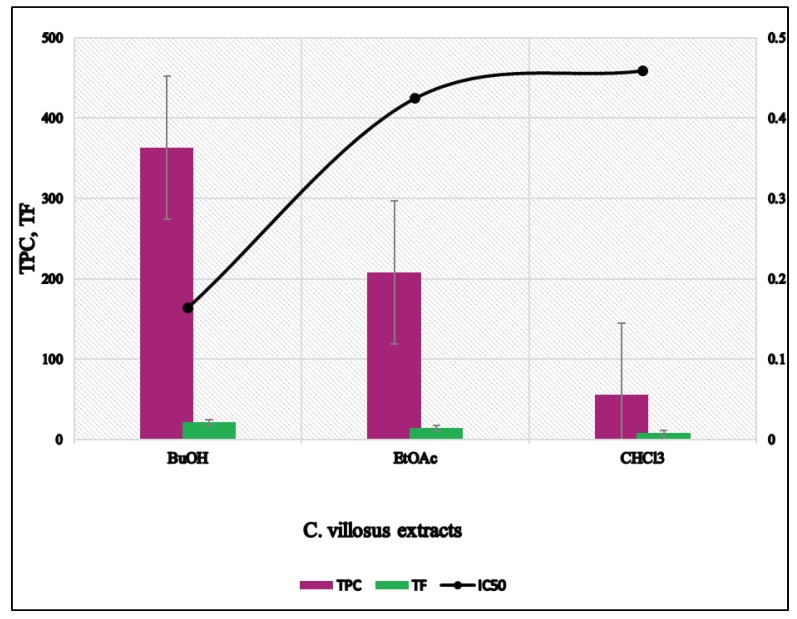
Comparison between total phenolic and total flavonoid content (TPC and TF, respectively) and DPPH (IC_50_) data in different extracts of *C. villosus.*

The relationship between total phenolic content and total flavonoid and antioxidant activity using DPPH assay of different extracts is shown in Figure 3 and Figure 4, respectively. Regression analysis showed that phenolic compounds contributed to about 74% (*R*^2^ = 0.744, *p*< 0.05) of radical scavenging properties in the extracts of *C. villosus* (Figure 4). Similarly, flavonoid compounds contributed to about 74% (*R*^2^ = 0.736, *p* < 0.05) of antioxidant activity in the extracts (Figure 4). Figure 5 shows the comparison between total phenolic and total flavonoid contents (TPC and TF, respectively) and radical scavenging potential (DPPH) expressed in (IC_50_) data in different extracts of *C. villosus.* Figure 5 also shows that the *n*-BuOH extract exhibited the highest radical scavenging potential (DPPH) expressed in (IC_50_). Hence, a high phenolic content is an important factor to determinate the antioxidant activity. This result is in agreement with previous studies, reporting that the phenolic compounds significantly contribute to the antioxidant activity in different plant species [49].

In contrast to the antioxidant assays results, the *n*-BuOH extract from the *C. villosus* aerial parts showed weak anti-inflammatory activity for the inhibition of iNOS expression, with an IC_50_ value of 90 µg/mL. Whereas the EtOAc extract exhibited higher inhibition of iNOS with an IC_50_ value of 48 µg/mL. The increase in transcriptional activity of NF-κB in PMA-treated cells was not suppressed by the plant’s extracts. Among all tested compounds, compound 1 from the EtOAc extract showed good inhibition of iNOS with an IC_50_ value of 9 µg/mL. This compound showed lower inhibition of NF-κB activity with an IC_50_ value of 28 µg/mL. Previous studies also indicated that genistein (1) acts as anti-inflammatory agent [50]. This isoflavone has been reported to have inhibitory effects on iNOS expression and to inhibit the activation of nuclear factor-*κ*B (NF-*κ*B) [51,52]. Hence, the EtOAc extract could be a good source of phenolics with anti-inflammatory activity. Our results also showed that *n*-BuOH extract exhibited potent antitrypanosomal activity against *T. brucei* with an IC_50_ value of 7.99 µg/mL and an IC_90_ value of 12.61 µg/mL. Compound 4 that was isolated from this extract didn’t show an effect against *T. brucei.* Future examination of the polar components of *C. villosus,* shall determine the active components from the *n*-BuOH extract.

The opioid system consists of three receptors, mu, delta, and kappa, which are activated by endogenous opioid peptides (enkephalins, endorphins, and dynorphins). The endogenous cannabinoid system comprises lipid neuromodulators (endocannabinoids), enzymes for their synthesis and their degradation, and two well-characterized receptors, cannabinoid receptors CB1 and CB2 [53]. Evidence has suggested that the opioid system can regulate inflammatory responses in rodents [54]. Mastinou et al. [25], recently described the link between neuroinflammation and cannabinoid systems. The radioligand displacement affinity towards opioid and cannabinoid receptors were evaluated for the extracts and isolated compounds of *C. villosus*. The EtOAc extract exhibited low/moderate activity towards the CB1 and CB2 receptors (32.1% and 25.2% displacement) and moderate activity in the delta (δ) opioid receptor (31.3% displacement). Similarly, the *n*-BuOH extract was found to have moderate activity towards CB1, CB2, and delta (δ) opioid receptors (33.7%, 26.1% and 24.8% displacement, respectively). None of the isolated compounds showed activity towards cannabinoid nor opioid receptors.

## 5. Conclusions 

In conclusion, we reported the phenolic and flavonoid content, antioxidant, anti-inflammatory, antibacterial, antifungal, antimalarial, antitrypanosomal, antileishmanial, and cytotoxicity activities, in addition to the affinity towards cannabinoid and opioid receptors, of *C. villosus* aerial parts extracts and their isolated compounds. Our results showed that the *n*-BuOH extract had the highest phenolic and flavonoid content. Furthermore, *n*-BuOH extract produced a potent antitrypanosomal activity that makes it a promising source for the extraction of bioactive components with high activity against human African trypanosomiasis. EtOAc extract was found to exhibit moderate anti-inflammatory activity against iNOS, while the *n*-BuOH extract showed lower inhibitory effect against iNOS. Among isolated compounds, genistein, which isolated from the EtOAc extract, showed the highest anti-inflammatory activity. Further explorations of EtOAc extract could afford more potent anti-inflammatory agents. Although the EtOAc and *n*-BuOH extracts showed moderate activity towards CB1, CB2, and δ opioid receptors, these results encourage further exploration of *Cytisus* species and its isolated compounds to study their cannabinoid and opioid receptors activities.

## Figures and Tables

**Figure 1:**
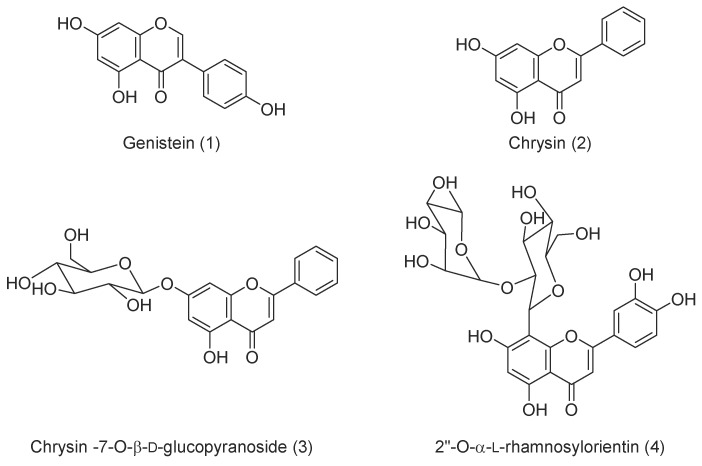
Compounds isolated of *Cytisus* *villosus* aerial parts.

**Table 1:** Total phenolic, flavonoid, and radical scavenging (DPPH) activity of *C. villosus* extracts.

Extract	Total Phenolic Content (mg GAE/g extract)	Total Flavonoid Content (mg QE/g extract)	DPPH Scavenging IC_50_ (mg/mL)	ARP = 1/IC_50_	mg AAE/g Extract=ARP Extract/ARP Ascorbic Acid
CHCl_3_	56.0 ± 2.50	7.70 ± 0.547	0.459 ± 0.002	2.180 ± 0.01	0.093 ± 0.004
EtOAc	208.0 ± 8.49	13.95 ± 1.058	0.425 ± 0.003	2.355 ± 0.018	0.100 ± 0.001
*n*-BuOH	363.0 ± 8.32	21.16 ± 1.022	0.164 ± 0.004	6.113 ± 0.157	0.268 ± 0.007
Ascorbic acid			0.043 ± 0.006	23.761 ± 3.257	

Values expressed are means ±SD of three parallel measurements. GAE. Gallic acid equivalents; QE. Quercetin equivalents; ARP. Antiradical power.

**Table 2:** Potential antioxidant activities of extracts and some isolated pure compounds.

	% Decrease in Oxidative Stress		
**Tested Organism**	Concentration (µg/mL)		
	1000	500	250
xtracts			
EtOAc	47	37	29
*n*-BuOH	49	39	**36**
Compounds			
1	NA	NA	NA
2	NA	NA	NA
3	NA	NA	NA
4	36	29	**28**
Quercetin 25 µM	77		

**Table 3:** Anti-inflammatory activities of extracts and some isolated compounds of *C. villosus*.

Tested Organism	Inhibition of iNOS IC_50_ (µg/mL)	Inhibition of NF-kB IC_50_ (µg/mL)	IC_50_ SP-1
Extracts			
EtOAc	48	NT	NT
*n*-BuOH	90	NT	NT
compounds			
1	**9**	28	NA
2	>25	38	NA
3	20	NA	NA
4	NA	NA	NA
Parthenolide	0.2	1.63	

NA = no activity at 25 or 100 µg/mL for pure compounds and extracts, respectively. NT = not tested.

**Table 4:** Antiprotozoal activity of extracts and some isolated compounds of *C. villosus*.

Tested Organism	*L. donovani* Promastigote IC_50_ (μM)	*L. donovani* Promastigote IC_90_ (μM)	*L. donovani* Amastigote IC_50_ (μM)	*L. donovani* Amastigote IC_90_ (μM)	*L. donovani* Amastigote/THP1 IC_50_ (μM)	*L. donovani* Amastigote/THP1 IC_90_ (μM)	*T. brucei* IC_50_ (μM)	*T. brucei* IC_90_ (μM)	THP1 Cytotoxicity IC_50_ (μM)	THP1 Cytotoxicity C_90_ (μM)
Extracts										
EtOH	>20	>20	>20	>20	>20	>20	19.48	>20	>20	>20
BuOH	>20	>20	>20	>20	>20	>20	7.99	12.61	>20	>20
Compounds										
3	>10	>10	>10	>10	>10	>10	>10	>10	>10	>10
4	>10	>10	>10	>10	>10	>10	>10	>10	>10	>10
Amphotericin B	0.136	0.215	0.211	0.374	0.188	0.421	NT	NT	>2	>2
Pentamidine	1.478	2.382	9.581	>10	1.157	5.587	0.001	0.002	>10	>10
DFMO	NT	NT	NT	NT	NT	NT	3.634	8.804	NT	NT

**Table 5:** Antimicrobial activity of extracts and certain isolated compounds of *C. villosus*

	% Growth Inhibition ^1,2^/IC_50_ μg/mL								
	Anti-Fungal				Anti-Bacterial				
Extract/ Compound	*C. albicans*	*C. glabrata*	*C. krusei*	*A. fumigatus*	*C. neoformans*	*S. aureus*	MRSA	*E. coli*	*P. aeruginosa*
*n*-BuOH	9	40	0	2	0	0	0	14	9
EtOAc	9	11	2	4	0	3	0	12	5
2	>20	NT	NT	NT	>20	>20	NT	>20	>20
3	>20	NT	NT	NT	>20	>20	NT	>20	>20
4	>20	NT	NT	>20	>20	>20	>20	>20	>20
AMB	100	NT	NT	93	100	NT	1	0	0
CIPRO	0	NT	NT	8	0	NT	0	100	96

Concentration: 50 μg/mL. ^1^ Samples showing % Growth Inhibition <50 are considered inactive; ^2^ Samples showing % Growth Inhibition >50 in any organisms are confirmed in secondary assay. Ciprofloxacin (CIPRO) and Amphotericin (AMB) = positive controls. Pure compounds that have an IC_50_ of ≤7 μg/mL in the secondary assay proceeded to the tertiary assay.

**Table 6:** Antimalarial activity of *C. villosus* extracts.

Tested Organism	%Inhibition		
Extract	*P. falciparum* (D6 Clone)	*P. falciparum* (W2 Clone)	Concentration ng/mL
BuOH	0	NT	158667
EtOAc	0	NT	158667
CQ	100	NT	79.3

CQ: Chloroquine (Positive Control).

**Table 7:** Antimalarial activity (IC_50_ values are in ng/mL) of compound.

	IC_50_	SI	IC_50_	SI	IC_50_	
CQ	˂26.0	>9	116	>2.1	>238	238-26.4
2	>4760	1	>4760	1	>4760	4760-528.9
3	>4760	1	>4760	1	>4760	4760-528.9

CQ: Chloroquine (Positive Control); SI: selectivity index (IC_50_ for cytotoxicity/IC_50_ for antimalarial activity).

**Table 8:** Cytotoxic activity of *C. villosus* extracts and isolated pure compounds

Cytotoxicity (IC_50_ µg/mL)						
Extract/Compound	SK-MEL	KB	BT-549	SK-OV-3	LLC-PK1	Vero
EtOAc	NA	NA	NA	NA	NC	NC
BuOH	NA	NA	NA	NA	NC	NC
3	NA	NA	NA	NA	NC	NC
doxorubicin	0.8	1.3	0.9	2	1.2	NC

IC_50_ is the concentration that affords 50% inhibition of cell growth. SK-MEL: Human malignant melanoma; KB: Human epidermoid carcinoma; BT-549: Human ductal carcinoma; SK-OV-3: Human ovary carcinoma; LLC-PK-1: Pig kidney epithelial cells; Vero: African green monkey kidney cell line. NA = No activity at 100 μM. NC = Not cytotoxic.

**Table 9:** Displacement radioligand assay for human opioid receptors (Subtypes δ, κ, and μ) and cannabinoid receptors (Subtypes CB_1_ and CB_2_) of *C. villosus.*

	Cannabinoid Receptors (%)		Opioid Receptors (%)		
Extract/Compound	CB_1_	CB_2_	δ	κ	μ
EtOAc	32.1	25.2	31.3	5.7	2.8
*n*-BuOH	33.7	26.1	24.8	10.3	5.7
2	7.7	0.8	8.7	12.8	12.2
naloxone			106.4	101.6	97.0
CP 55,940	104.3	102.6			

Naloxone and CP 55,940 = Positive controls.

## Supplementary Materials

The following are available online at https://www.mdpi.com/2218-273X/9/11/732/s1, Figures S1–S12, spectroscopic data for the isolated compounds.
