# Supplementary material for: Total Phenolic and Flavonoid Content and Biological Activities of Extracts and Isolated Compounds of Cytisus villosus Pourr
Source: Biomolecules. 2019 Nov 13;9(11):732. doi: 10.3390/biom9110732 (PMC6920997; doi:10.3390/biom9110732)
Supplement: Supplementary file 1 [file biomolecules-09-00732-s001.pdf]

SUPPLEMENTARY MATERIAL

Article

Total Phenolic and Flavonoid Content and Biological Activities of Extracts and Isolated Compounds of *Cytisus villosus* Pourr.

Farida Larit [1,2,*], Francisco León [1,3], Samira Benyahia [4] and Stephen J. Cutler [1,5]

[1] Department of BioMolecular Sciences, School of Pharmacy, University of Mississippi, University, city, state abbreviation, MS 38677-1848, USA
[2] Département de Chimie, Faculté des Sciences Exactes, Université des Frères Mentouri Constantine 1, Constantine, Route d'Aine El Bey 25000, Constantine, Algeria
[3] Department of Medicinal Chemistry, College of Pharmacy, University of Florida, Gainesville, FL 32610, USA
[4] Laboratoire de Synthèse Organique, Modélisation et Optimisation des Procèdes (LOMOP), Université Badji Mokhtar, Faculté des Sciences, Département de Chimie, 23000 Annaba, Algeria; samira.benyahia13@gmail.com
[5] College of Pharmacy, University of South Carolina, Columbia, SC 29208, USA
* Correspondence: laritfarida@umc.edu.dz; Tel.: +213-664-79-89

Received: 19 October 2019; Accepted: 11 November 2019; Published: date

List of Contents

Figure S1. ^{1}H NMR spectrum of compound 1

Figure S2. ^{13}C NMR spectrum of compound 1

Figure S3. HRESIMS (-) for compound 1

Figure S4. ^{1}H NMR spectrum of compound 2

Figure S5. ^{13}C NMR spectrum of compound 2

Figure S6. Negative HRESIMS for compound 2

Figure S7. ^{1}H NMR spectrum of compound 3

Figure S8. ^{13}C NMR spectrum of compound 3

Figure S9. Negative HRESIMS for compound 3

Figure S10. ^{1}H NMR spectrum of compound 4

Figure S11. ^{13}C NMR spectrum of compound 4

Figure S12. Negative HRESIMS for compound 4

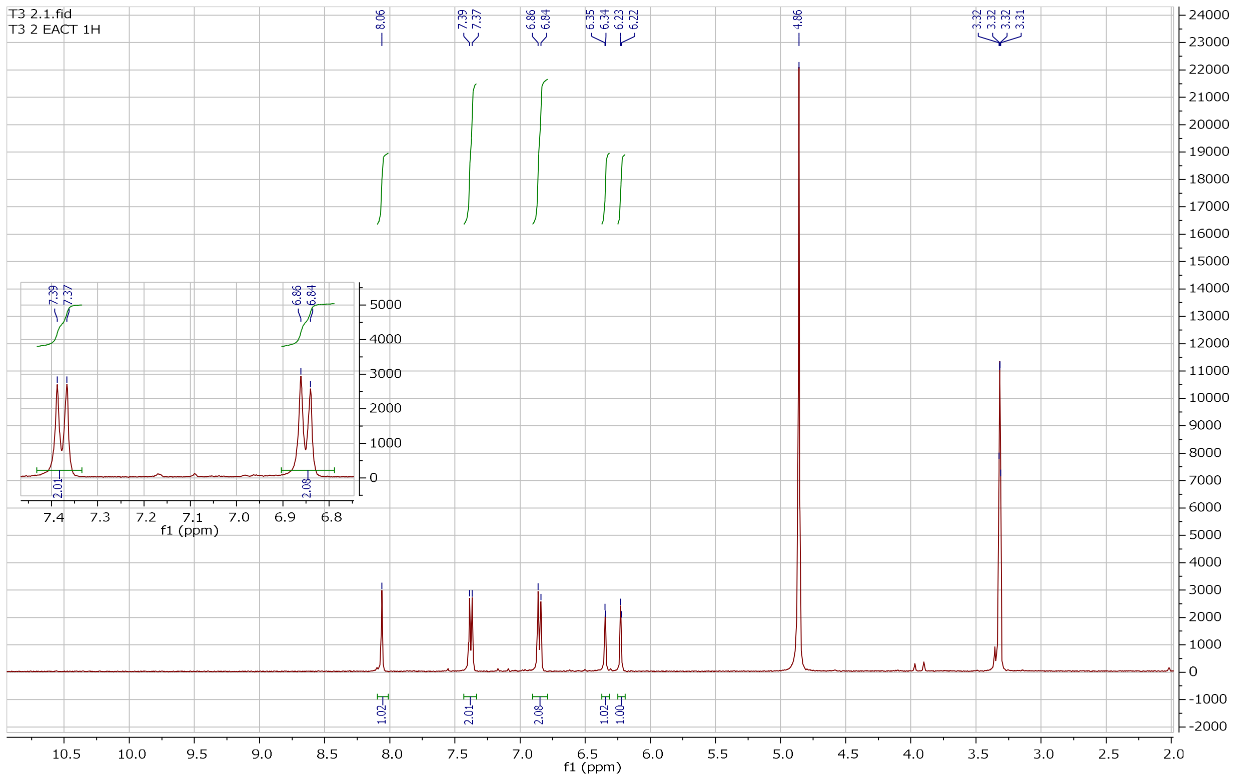

Figure S1: ^{1}H NMR spectrum of compound 1

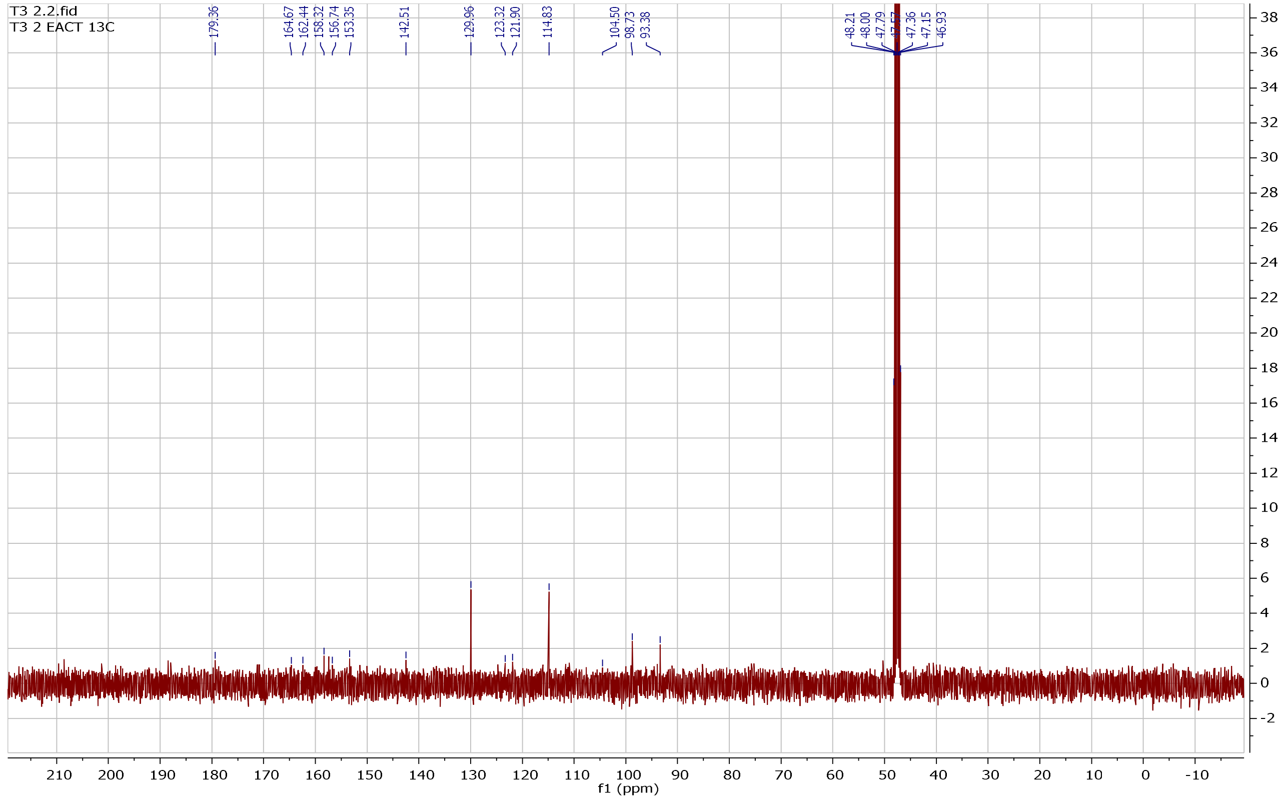

Figure S2: ^{13}C NMR spectrum of compound 1

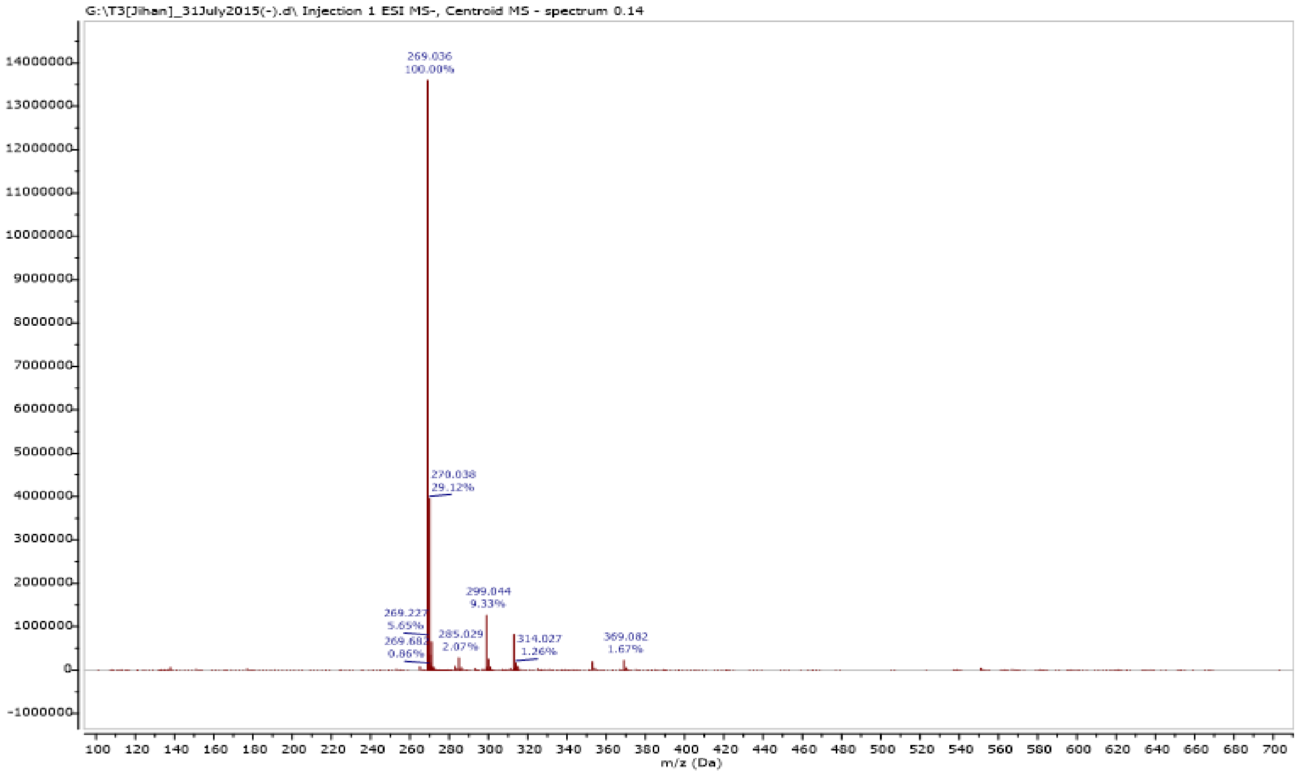

Figure S3: Negative HRESIMS for compound 1

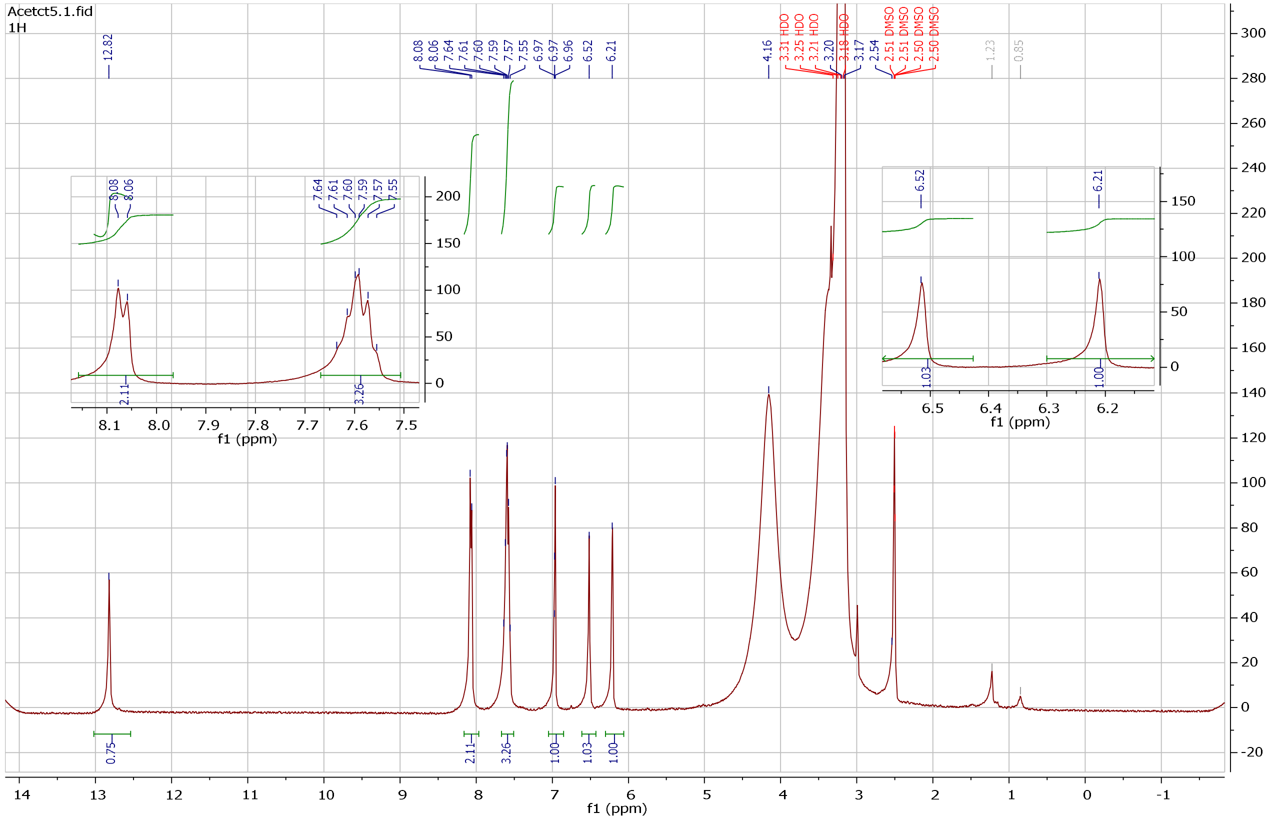

Figure S4: ^{1}H NMR spectrum of compound 2

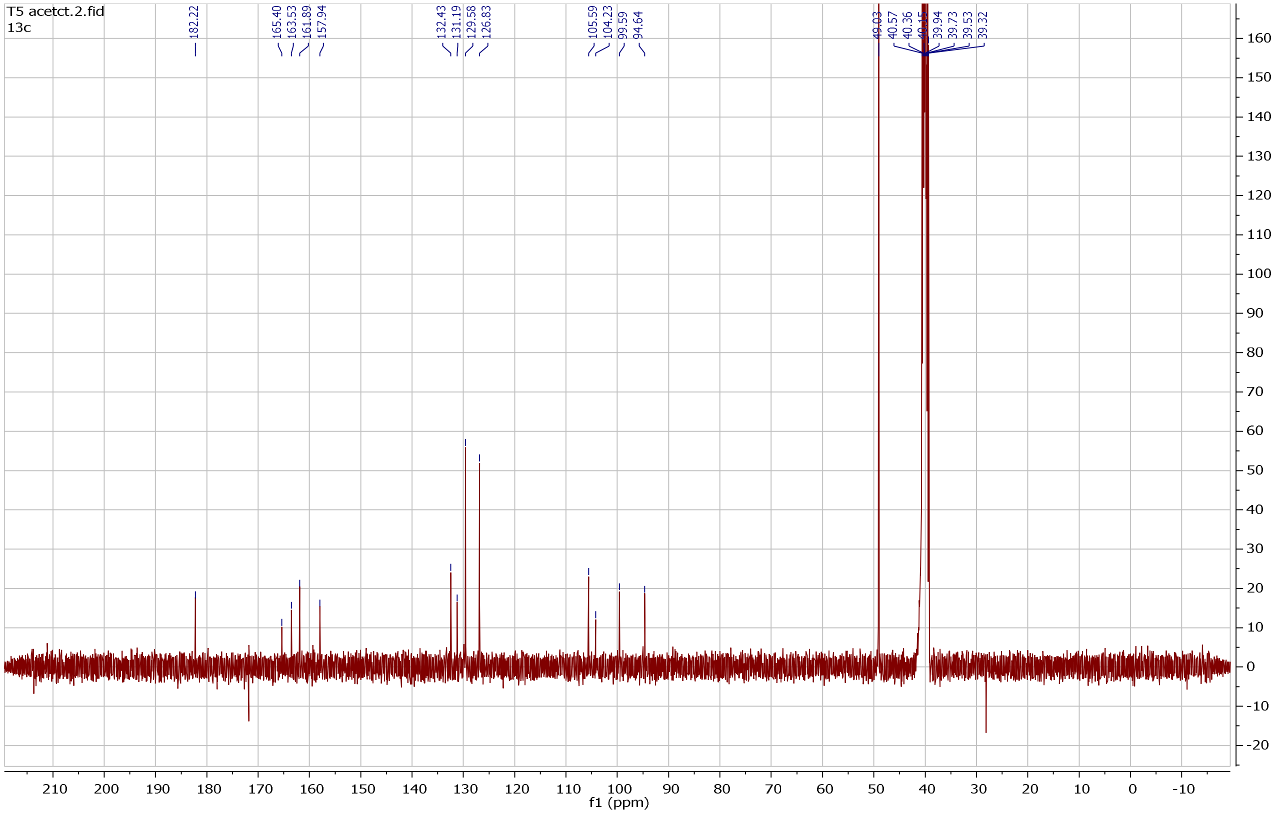

Figure S5: ^{13}C NMR spectrum of compound 2

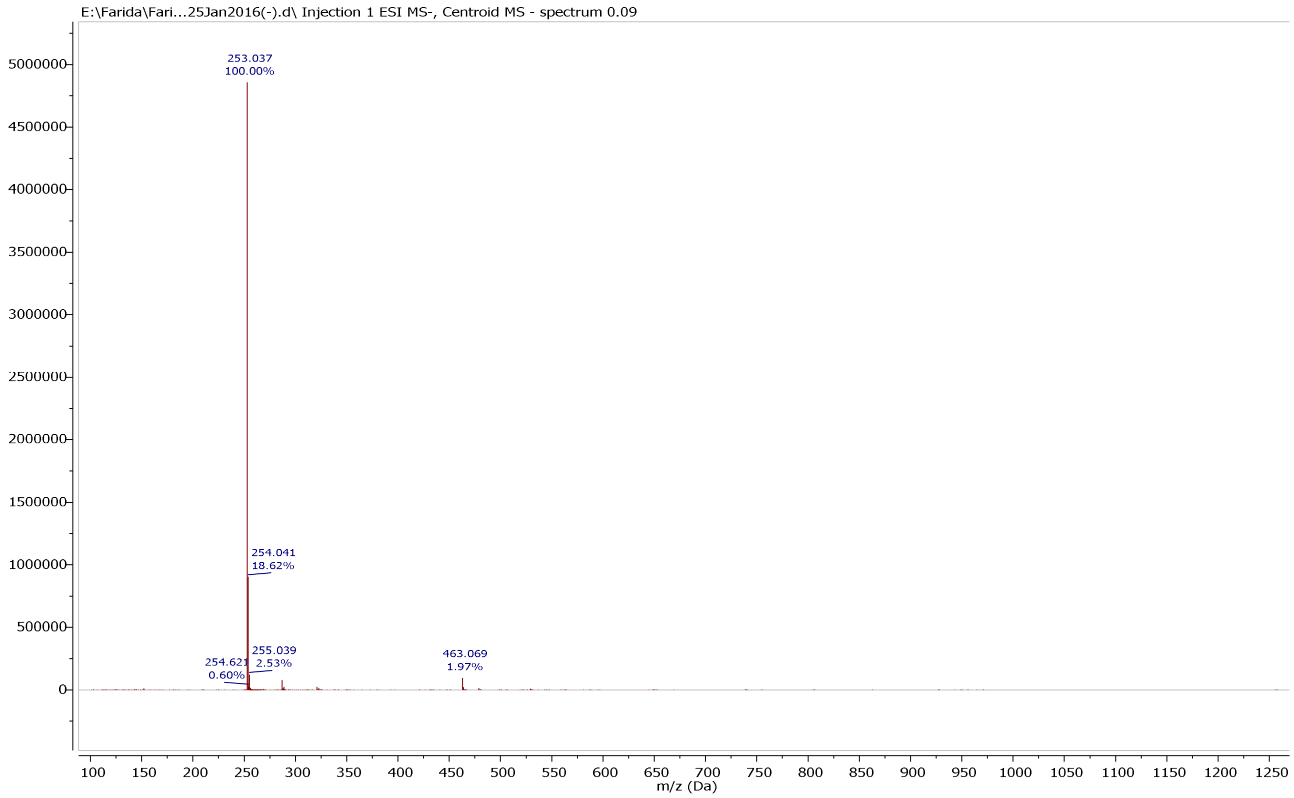

Figure S6: Negative HRESIMS for compound 2

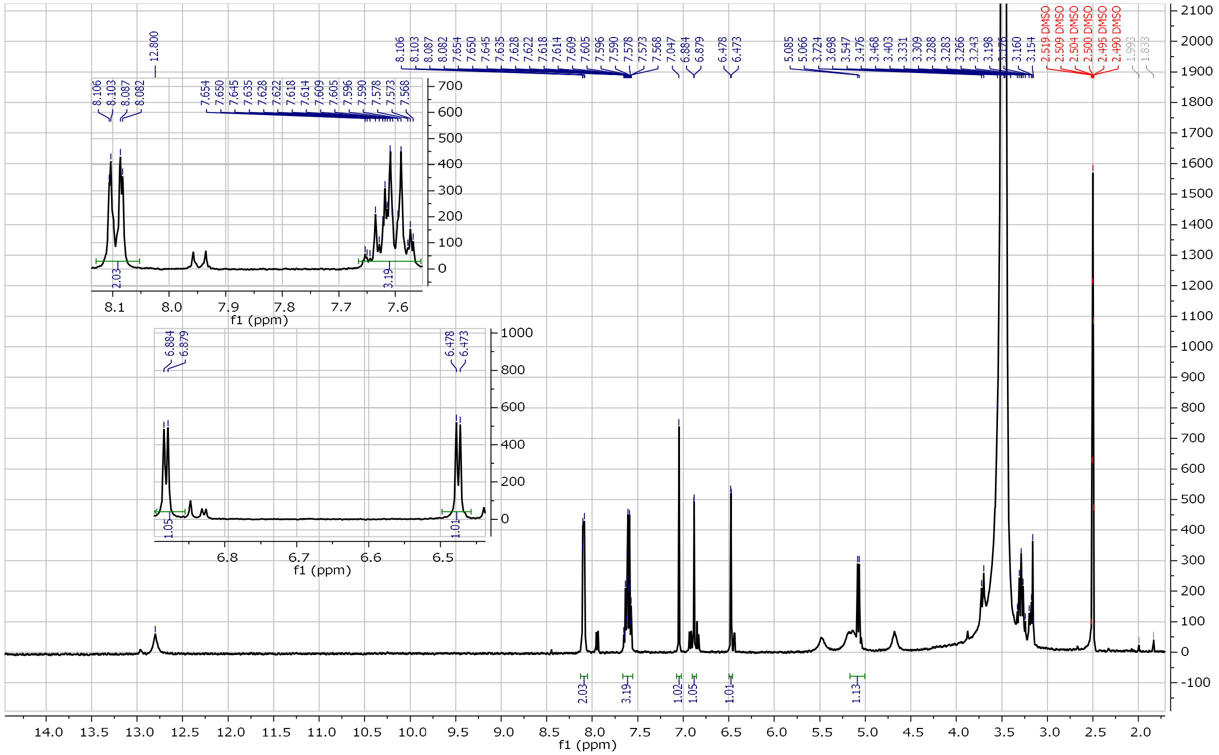

Figure S7: ^{1}H NMR spectrum of compound 3

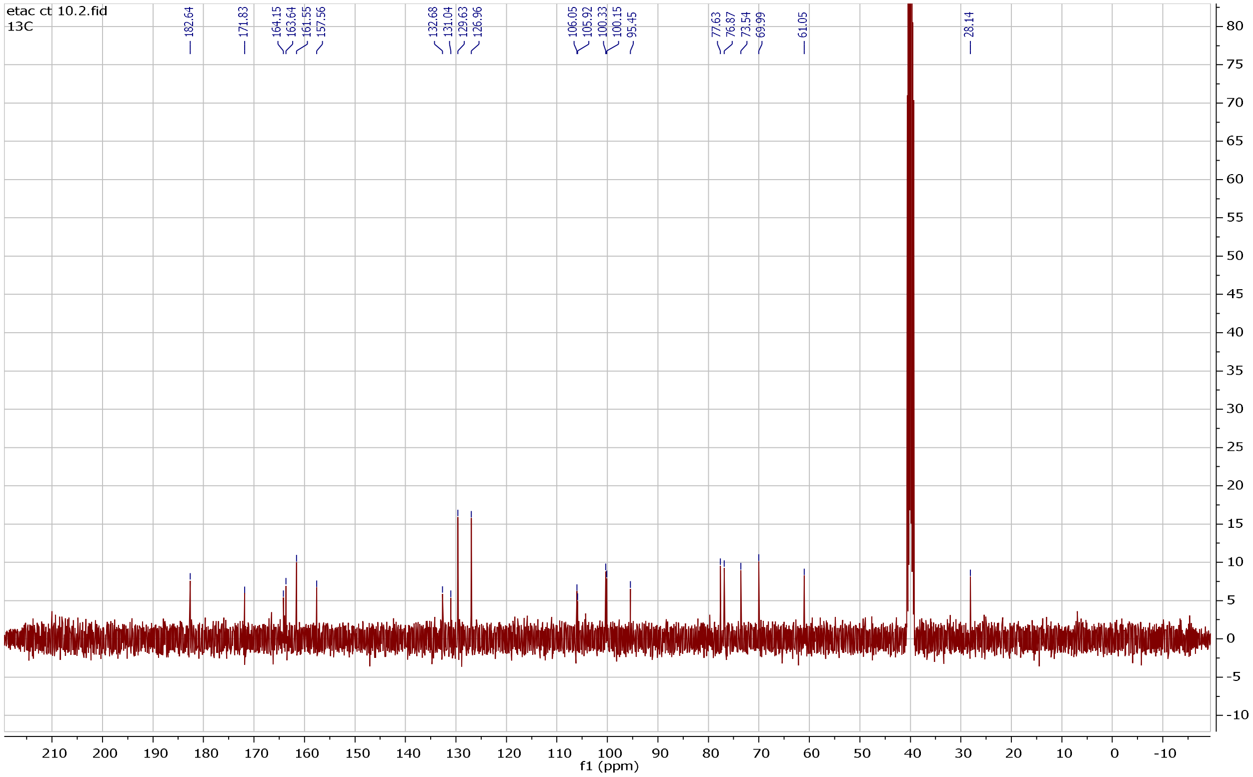

Figure S8: ^{13}C NMR spectrum of compound 3

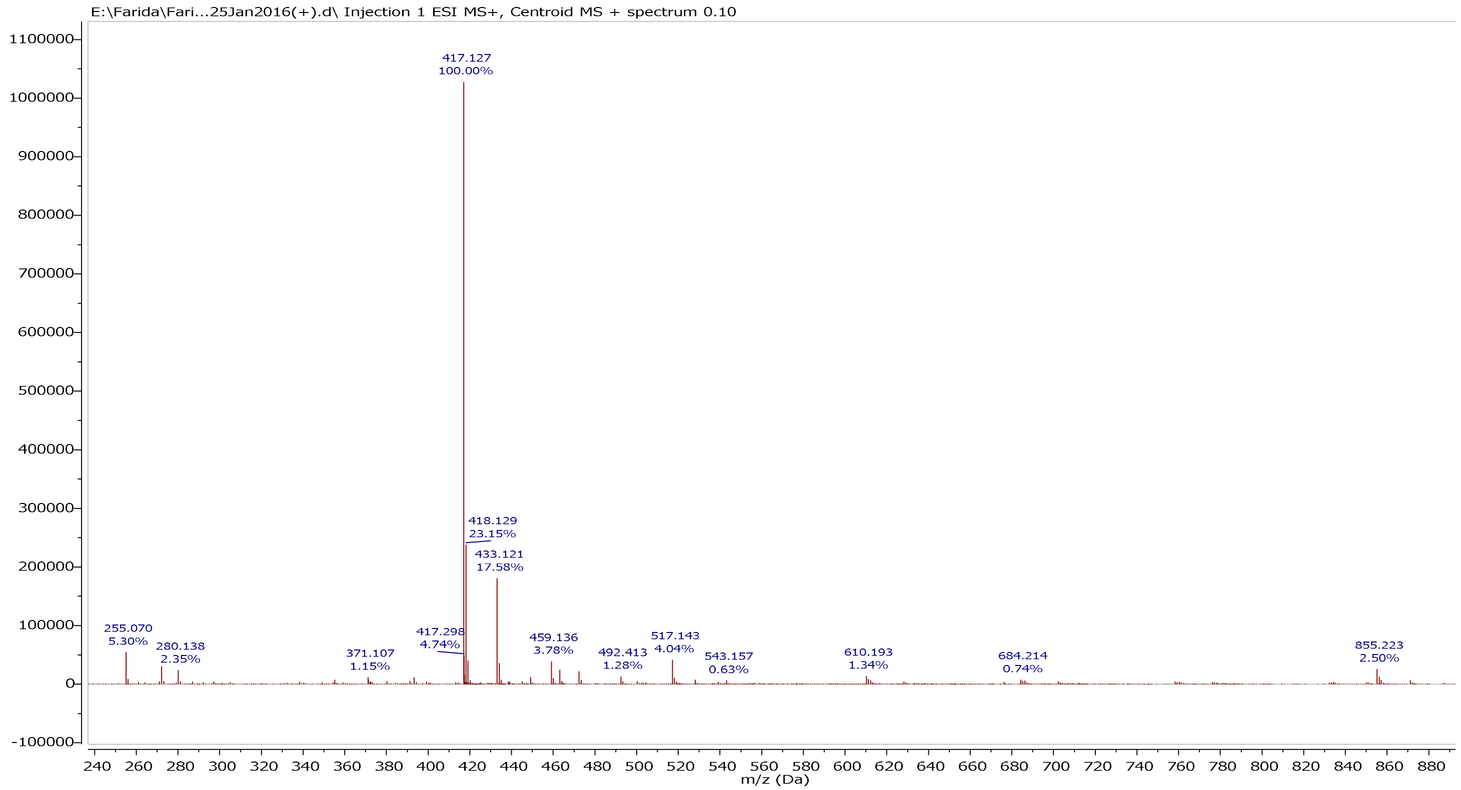

Figure S9: Negative HRESIMS for compound 3

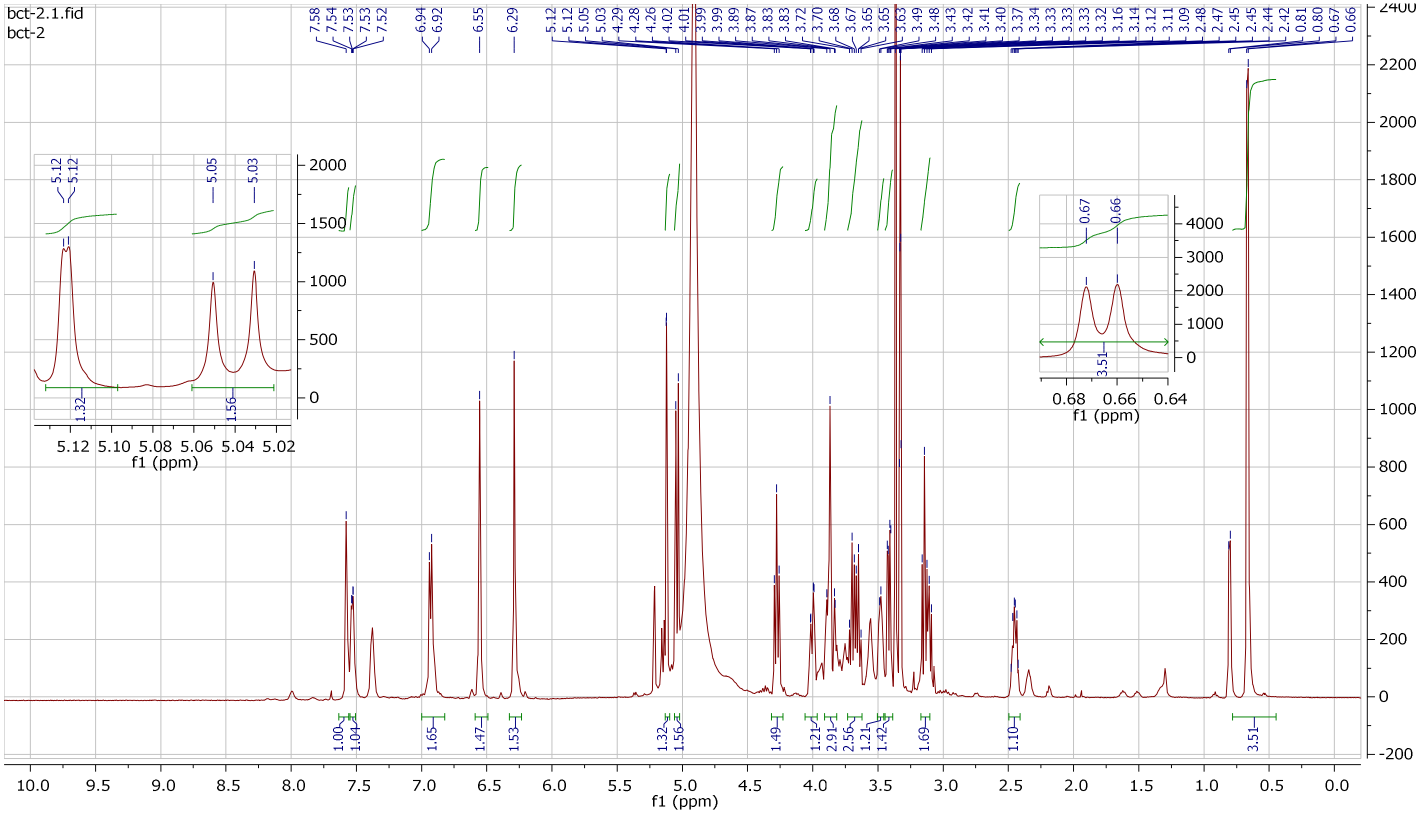

Figure S10: ^{1}H NMR spectrum of compound 4

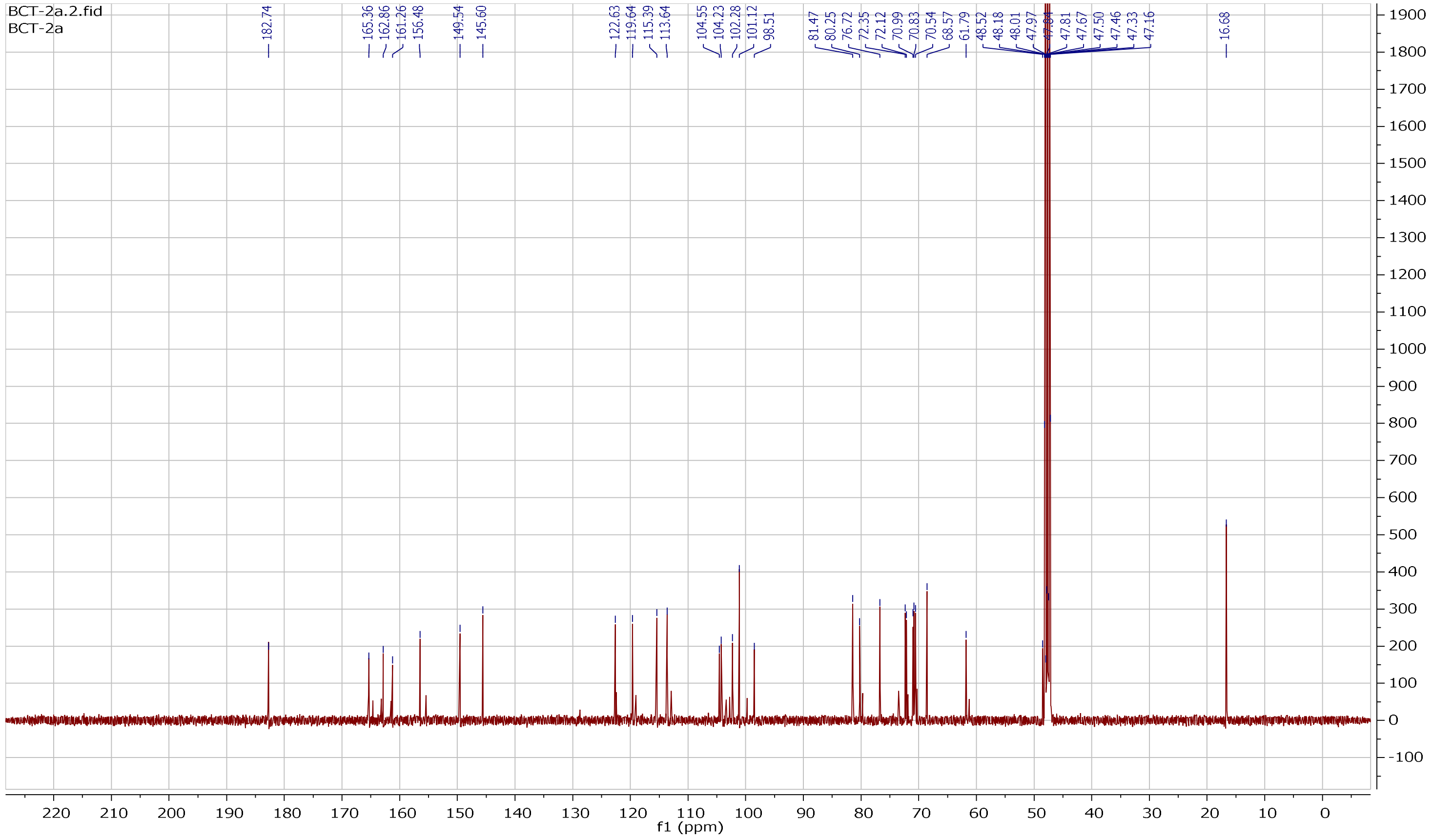

Figure S11: ^{13}C NMR spectrum of compound 4

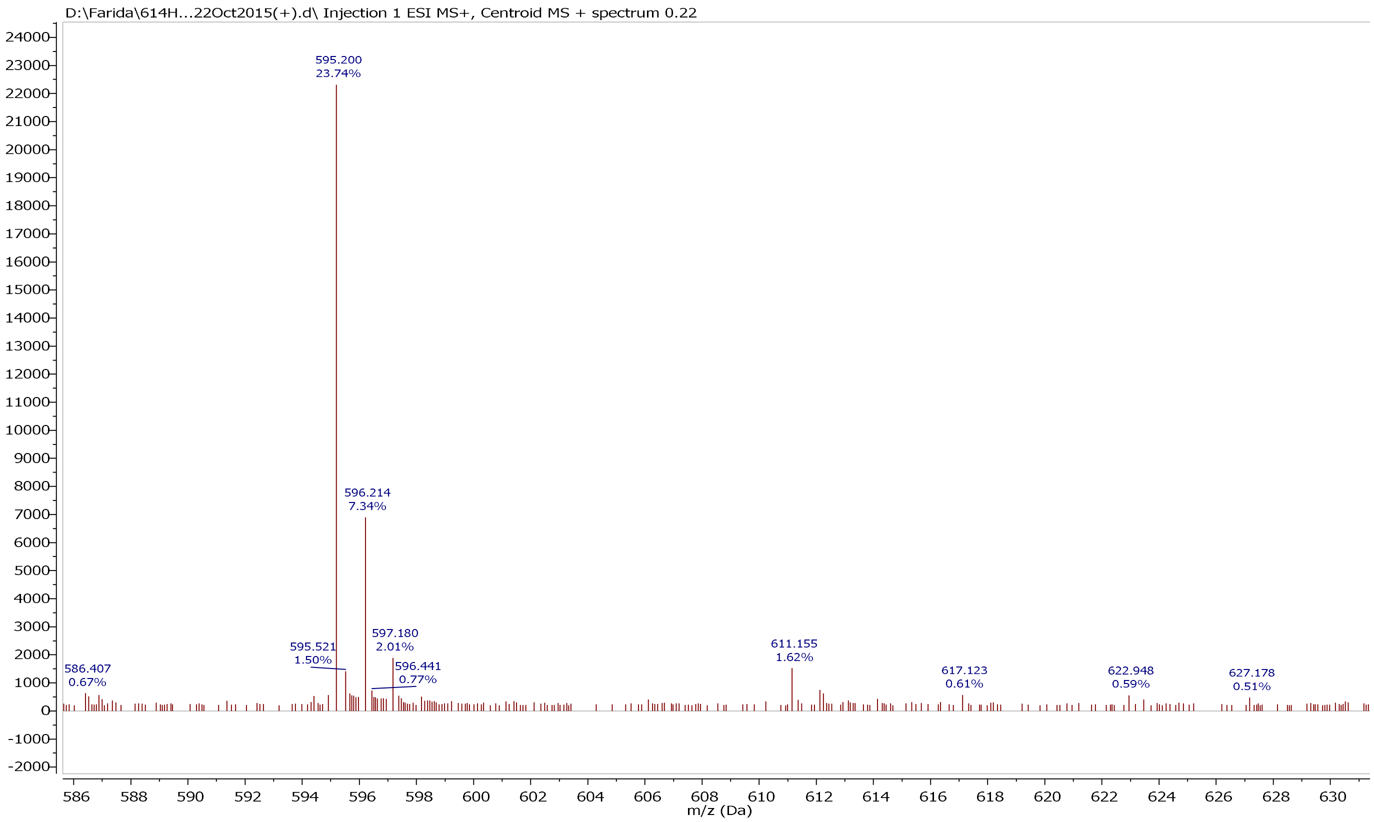

Figure S12: Negative HRESIMS for compound 4
